# Pituitary adenylate cyclase–activating polypeptide (PACAP-38) plays an inhibitory role against inflammation induced by chemical damage to zebrafish hair cells

**DOI:** 10.1371/journal.pone.0198180

**Published:** 2018-06-01

**Authors:** Natalia Kasica-Jarosz, Piotr Podlasz, Jerzy Kaleczyc

**Affiliations:** 1 Department of Animal Anatomy, Faculty of Veterinary Medicine, University of Warmia and Mazury in Olsztyn, Olsztyn, Poland; 2 Department of Pathophysiology, Forensic Veterinary and Administration, Faculty of Veterinary Medicine, University of Warmia and Mazury in Olsztyn, Olsztyn, Poland; Universite de Rouen, FRANCE

## Abstract

Pituitary adenylate cyclase–activating polypeptide (PACAP-38) is a common neuropeptide exerting a wide spectrum of functions in many fields, including immunology. In the present study, 5-day post-fertilization (dpf) zebrafish larvae of three diverse genetic lines [transgenic lines Tg(MPX:GFP) with GFP-labelled neutrophils and Tg(pou4f3:GAP-GFP) with GFP-labelled hair cells and the wild-type Tuebingen] were used to investigate an inhibitory role of PACAP-38 in inflammation associated with damaged hair cells of the lateral line. Individuals of each genetic line were assigned to four groups: (1) control, and those consisting of larvae exposed to (2) 10 µM CuSO_4_, (3) 10 µM CuSO_4_+100 nM PACAP-38 and (4) 100 nM PACAP-38, respectively. Forty-minute exposure to CuSO_4_ solution was applied to evoke necrosis of hair cells and consequent inflammation. The inhibitory role of PACAP-38 was investigated *in vivo* under a confocal microscope by counting neutrophils migrating towards damaged hair cells in Tg(MPX:GFP) larvae. In CuSO_4_-treated individuals, the number of neutrophils associated with hair cells was dramatically increased, while PACAP-38 co-treatment resulted in its over 2-fold decrease. However, co-treatment with PACAP-38 did not prevent hair cells from extensive necrosis, which was found in Tg(pou4f3:GAP-GFP) individuals. Real-Time PCR analysis performed in wild-type larvae demonstrated differential expression pattern of stress and inflammation inducible markers. The most significant findings showed that CuSO_4_ exposure up-regulated the expression of *IL-8*, *IL-1β*, *IL-6* and *ATF3*, while after PACAP-38 co-treatment expression levels of these genes were significantly decreased. The presence of transcripts for all PACAP receptors in neutrophils was also revealed. *Adcyap1r1a* and *vipr1b* appeared to be predominant forms. The present results suggest that PACAP-38 should be considered as a factor playing an important regulatory role in inflammatory response associated with pathological processes affecting zebrafish hair cells and it cannot be excluded that this interesting property has more universal significance.

## Introduction

Pituitary adenylate cyclase–activating polypeptide (PACAP-38) is a pleiotropic neuropeptide, with known protective and anti-apoptotic functions [1–6]. In recent decades, PACAP-38 has been also classified as an anti-inflammatory factor which regulates inflammatory responses via influencing both anti- and pro-inflammatory mediators. PACAP-38 exerts its role in the inflammation process through three receptors, VPAC1, VPAC2 and PAC1. It has been already demonstrated that PACAP-38 and its receptors are evolutionarily well-conserved among species, including mammals or teleost fish and are present in their immune systems [7, 8]. The anti-inflammatory action of PACAP-38 is multi-faceted. It regulates production of pro-inflammatory macrophage-derived mediators, such as TNF-α, IL-6, IL-12 [7] or anti-inflammatory effectors like IL-10 [9,10]. It has also been demonstrated that PACAP-38 modulates many macrophage functions, stimulating migration, adherence or phagocytosis [11,12]. Moreover, the effects of PACAP-38 on lymphocyte function, survival and differentiation have been broadly discussed [7]. Comparatively few studies have dealt with the influence of PACAP-38 on neutrophils. The only available contributions concerning humans and mice have, unfortunately, reported the completely opposite effects. Kinhult et al. (2001) [13] and Martinez et al. (2005) [14] found that administration of PACAP-38 inhibits neutrophil chemotaxis, while Kim et al. (2006) [15] revealed that a shorter form of this peptide—PACAP-27 stimulates neutrophil migration. In contrast, neutrophils incubated with PACAP-38 exhibited a marked increase in the expression of cell surface CD11b, CD63 and CD66b markers, indicating its role in granulocyte activation [16]. This suggests that different pathways can mediate chemotaxis and cellular activation and that further studies are needed.

The use of zebrafish (*Danio rerio*) in biomedical research is invaluable and in recent years has gradually expanded into the field of immunology. The most preferable stage of the zebrafish lifetime in both medical [17] as well as veterinary studies [18], is the early life period. Immunological studies on the individuals of early life stages have dealt with the only form of immune system functioning in this phase of ontogenesis, the innate immune system, which starts developing already at the 1st day post fertilization (dpf) [19,20], thus, much earlier before the acquired immune system does. Several studies have proven the remarkable similarity of zebrafish immune system to that in humans and have revealed that almost all cell types of the human immune system have zebrafish counterparts [21]. Unfortunately, the role of PACAP-38 and the molecular basis of its action in zebrafish immune system still remains to be uncovered.

Our previous contributions have demonstrated that PACAP-38 plays an anti-apoptotic role in oxidative stress-damaged zebrafish hair cells, which is very promising information in the context of (as far as) working out suitable models for studying PACAP-38 properties in ototoxicity screening assays [6]. The zebrafish lateral line (posterior and anterior) consists of individual sense organs called neuromasts, composed of hair cells. It has been proven that zebrafish hair cells greatly resemble those in the mammalian inner ear [22], making the zebrafish an ideal model for auditory system investigations. Many factors and signaling pathways activated by inflammation are involved in the regulation of cell apoptosis [23]. Therefore, we have assumed the hypothesis that PACAP-38 plays an anti-inflammatory role in inflammation induced by copper damage to zebrafish hair cells.

Considering the above-mentioned issues, the present study was designed to establish the role of PACAP-38 in non-invasive inflammation induced by chemical damage to zebrafish hair cells. Based on the previous information, it was assumed that the ongoing inflammatory process is indicated by the migration of neutrophils from the caudal hematopoietic tissue (CHT) or the posterior blood island (PBI) to the neuromasts [24]. The aim was to investigate how PACAP-38 influences neutrophil migration and whether it modulates the gene expression profile of chosen immune and stress response markers [activating transcription factor 3 (*ATF3*), interleukin 1β (*IL-1β*), interleukin 6 (*IL-6*), interleukin 8 (*IL-8*), interleukin 10 (*IL-10*), Macrophage receptor MARCO (*MARCO*), tumor necrosis factor (TNFα)]. Because IL-8 is known as a neutrophil chemotactic factor, more account of this chemokine was taken and it was decided to focus on the gene expression of IL-8 receptors, C-X-C motif chemokine receptor 1 (CXCR1) and C-X-C motif chemokine receptor 2 (CXCR2). It was also important to clarify whether PACAP-38 exerts its effect indirectly by influencing the production of the cytokines and transcription factors or by direct impact on immune cells. Therefore, the goal was to demonstrate the presence of PACAP receptors on neutrophils to shed some additional light on the mechanism involved in the investigated processes. The data obtained should provide new insights into toxicological and molecular mechanisms of PACAP-38 anti-inflammatory effect and, thus, hopefully broaden the field of research on inflammation using zebrafish as an animal model.

## Materials and methods

### Animals

The study involved 5-day post-fertilization (dpf) zebrafish larvae of three diverse genetic lines: transgenic lines Tg(MPX:GFP) and Tg(pou4f3:GAP-GFP) and the wild-type Tuebingen, as well as 9-month-old fish of Tg(MPX:GFP) line. Tg(MPX:GFP) zebrafish transgenic line (kindly gifted from the Institute of Biology, Leiden University, Netherlands) was used for *in vivo* investigation of neutrophil migration towards damaged neuromasts in larvae and to isolate neutrophils from kidneys from adult fish, respectively. The Tg(MPX:GFP) line carries myeloperoxidase promoter, driving the expression of GFP in myeloid leukocytes (mostly neutrophils). Necrosis assessment was accomplished in the Tg(pou4f3:GAP-GFP) zebrafish transgenic line (kindly gifted from the University of Sheffield, United Kingdom) which carries POU class 4 homeobox 3 promoter driving expression of green fluorescent protein (GFP) in hair cells. To investigate changes in the expression profile of genes encoding chosen inflammatory markers, the wild-type Tuebingen strain (kindly gifted from the Nüsslein-Volhard Lab, Max-Planck-Institut für Entwicklungsbiologie in Tübingen, Germany) was used. The adult fish were maintained in 8l tanks in a flow system at 28°C with a 14h light:10h dark photoperiod, and fed three times daily *ad libitum* with dry food and *Artemia sp*. naupli. The embryos were maintained in an embryo solution (E3 medium) (5 mM NaCl, 0.17 mM KCl, 0.33 mM CaCl_2_, 0.33 mM MgSO_4_) and kept in an incubator at 28.5°C and 14h light:10h dark photoperiod without feeding until 5 dpf. The individuals used in the study were anesthetized by placing them in a tricaine methanesulfonate (MS-222) solution and euthanized by an overdose of MS-222, respectively.

All fish are housed in the fish facility of the Laboratory of Genomics and Transcriptomics, University of Warmia and Mazury in Olsztyn, Olsztyn, Poland, which was built according with the local animal welfare standards. All animal procedures were performed in accordance with Polish and European Union animal welfare guidelines. According to the European Directive 2010/63/EU and Polish law regulations O.J. of 2015, item 266, all procedures performed in the present study include the use of early life-stage zebrafish and euthanasia for the purpose of organ dissection do not require Ethic Committee permissions.

### The study design

All experiments were performed on 5 dpf larvae. For this purpose, the individuals of each genetic line were randomly assigned to 4 groups: (1) a control group including larvae which were incubated in embryo solution (E3 medium); (2) a group of larvae exposed to 10 µM CuSO_4_ for 40 min; (3) to investigate the ameliorative role of PACAP-38, a group of larvae were incubated with a mixture of 10 µM CuSO_4_ and 100 nM PACAP-38 for 40 min following 1 hour pre-incubation with 100 nM PACAP-38 only; (4) to check if PACAP-38 has any influence on the inflammatory process itself, a group of larvae were incubated with 100 nM PACAP-38 only. PACAP-38 was synthesized by the solid-phase technique utilizing ^t^Boc chemistry [25] as described previously [26]. The sequence of PACAP-38 used in the current study referred to mammalian PACAP-38 demonstrating 80% homology between the zebrafish and human peptide sequence and the zebrafish receptor binding site sequence corresponded to that of humans in almost 100% [6].

To demonstrate the presence of PACAP receptors in neutrophils we used 9-month-old fish. For this purpose, we decided to acquire a clear neutrophil population from kidneys, because this organ is commonly used as the source of neutrophils in the zebrafish. Fluorescence activated cell sorting (FACS) (details of the method are described in Materials and methods section) allowed obtaining the cell suspension of 98% purity. Therefore, to establish which receptors are specific for neutrophils and demonstrate their relative level of expression, we compared the results obtained in neutrophils to those gained from unsorted kidney tissues as well as whole 5 dpf larvae.

### Assessment of chemically induced neutrophil migration to damaged hair cells

**Fig 1:**
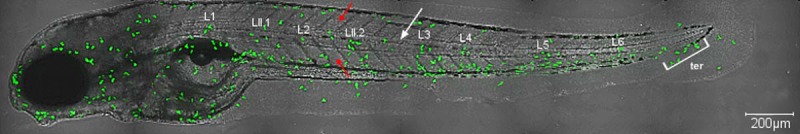
A photograph illustrating the neutrophil counting area. 5 dpf Tg(MPX:GFP) transgenic zebrafish larvae after 40 min exposure to 10 µM CuSO4 presents the area were neutrophils were quantified (green dots; in the intact larvae only single neutrophils were observed). Both neutrophils associated with investigated neuromasts (L1, LII.1, L2, LII.2, L3, L4, L5 and L6) as well as those which did not adhere directly to the neuromasts (but were sparsely found within the area encircled by the notochord [white arrow]) were counted. Dorsal and ventral myotomes marked with red arrows and terminal neuromasts (ter) were excluded from the analysis. The larvae carried myeloperoxidase promoter driving the expression of green fluorescent protein (GFP) in myeloid leukocytes (mostly neutrophils). The visualization was accomplished using a Zeiss LSM-700 confocal microscope.

5dpf Tg(MPX:GFP) zebrafish divided into four groups according to the above description were used. Each group consisted of n = 15 individuals. The induction of inflammation in the posterior lateral line (PLL) neuromasts was based on already established dynamics of this process [24]. It was found that the resulting effects of the 40 min 10 µM CuSO_4_ exposure were not intensified within the next 20 minutes, which enabled counting neutrophils in each experimental group *in vivo* within a 10 minute period. Anesthetized (MS-222, Sigma Aldrich) larvae were mounted on slices in 3% methyl cellulose and the remaining lateral line neutrophils were counted under a LSM 700 confocal laser scanning microscope (Zeiss, Germany). The analysis involved all trunk neuromasts (L1, LII.1, L2, LII.2, L3, L4, L5 and L6) excluding the 3 terminal (ter) ones where migrated neutrophils were barely distinguishable from those in the caudal hematopoietic tissue (CHT), which is a natural site of their occurrence at this developmental stage. The quantification was restricted to the area defined by the notochord, excluding cells located within the ventral and dorsal myotomes (Fig 1). Therefore, we counted fluorescent neutrophils associated with each neuromast mentioned as well as those which did not adhere directly to the neuromasts, but they were sparsely found in the area encircled by the notochord. Following d’Alençon et al. (2010) [24] it was not necessary to label neuromasts, as their localization is conservative, so they always appear along the notochord.

### Assessment of hair cells necrosis

For the purpose of this experiment, 5 dpf Tg(pou4f3:GAP-GFP) larvae were used. The experimental conditions remained the same as described previously. Each group consisted of n = 15 individuals. After the treatments, the larvae were fixed in 4% paraformaldehyde (PFA) o/n in 4°C. The next day, they were rinsed three times in phosphate-buffered saline (PBS) and mounted on their side on slices in 50% and then in 80% glycerol. The visualization was accomplished using a LSM 700 confocal laser scanning microscope (Zeiss, Germany).

### Microscopy and visualization

The visualization and images were achieved using a LSM 700 confocal laser scanning microscope (Zeiss, Germany). The GFP driven by myeloperoxidase promoter in Tg(MPX:GFP) line and by POU class 4 homeobox 3 in Tg(pou4f3:GAP-GFP) was excited by a 488 nm laser. To obtain desirable quality of images, ×10 and ×20, ×40 objectives, z-stack tool, as well as tile scan tool (when necessary) were applied. Stacks of images were composed into one to obtain maximum intensity projection images with ZEN 2009 software (Zeiss, Germany).

### Whole kidney extraction and preparation of cell suspension

Adult Tg(MPX:GFP) fish were euthanized with an overdose (500 mg/l) of MS-222. Kidneys from 5 individual fish were carefully removed and collected into 1.5 ml Eppendorf tubes with 1X Ringer solution (116 mM NaCl, 2.6 mM KCl, 5 mM HEPES, pH 7.0). After dissection, the tissues were transferred on falcon 40 µm cell strainer and entirely wiped into a 50 ml sterile falcon tube with 2 ml of suspension media (1% calf serum, 0.8 mM CaCl2, 50 U/mL penicillin, 0.05 mg/mL streptomycin, DMEM). The wiping procedure enabled separating a cell suspension containing neutrophils. As the Tg(MPX:GFP) line possesses GFP-labeled neutrophils, with the use of the stereoscope it was possible to visually assess the quality of separation and confirm the presence of neutrophils in the obtained suspension. The cell suspension was then rapidly processed with fluorescence-activated cell sorter (FACS).

### Isolation of GFP-positive cells using fluorescence-activated cell sorter (FACS)

To separate and collect GFP-positive (GFP+) and GFP-negative (GFP-) fractions, the cell suspension was passed through a MoFlo ^TM^ XDP fluorescence activated cell sorter (FACS) (Beckam Coulter) equipped with a 488 nm air-cooled argon solid state laser and a standard filter setup. An IsoFlow™ solution (Beckman Coulter) was used as the sheath fluid; the pressure of the sheath fluid was 60 psi, and the nozzle size was 70 μm. To minimize RNA degradation and keep cells alive, the sorted cells were collected directly into sterile 5-mL polypropylene tubes with Dulbecco′s Modified Eagle′s Medium (DMEM) (Merck). Moreover, after acquiring 100,000 events the tube was transferred on ice and replaced by a new one. Flow rate and the concentration of samples were adjusted to keep the acquisition lower than 500 events/s. The sorting procedure was stopped after acquiring 500,000 GFP+ cells. The settings used for the sorting were determined empirically and are provided as supporting information (S1 File). Immediately after the sorting procedure we isolated RNA from both GFP+ cells as well as unsorted material derived from the kidney tissue.

### RNA extraction and reverse transcription

Gene expression analysis was performed in 5 dpf wild-type Tuebingen zebrafish strain, sorted cells and unsorted kidney tissue. Immediately following the treatments, the larvae of the control and each experimental group were pooled (n = 30), frozen and stored in -80°C. Total RNA was extracted from pooled frozen larvae using Total RNA Mini isolation kit (AA Biotechnology). All steps of isolation were assessed according to the respective manufacturer's protocols. Homogenization required for RNA isolation was made using TissueLyser II (Qiagen). With regard to the sorted GFP+ cells and unsorted kidney tissue, just after FACS they were spun and the DMEM (Merck) was drained off. Total RNA was extracted using RNeasy Mini Kit (QIAGEN) according to the respective manufacturer's protocols. The cDNA samples were synthesized from respective high quality matrix samples with equal RNA concentration for each sample using Maxima First Strand cDNA Synthesis Kit for RT-qPCR (Thermo Scientific). All steps of reverse transcription were assessed according to the manufacturer's protocols.

### Real-Time PCR

Real-time PCR was performed using SYBR Green in accordance with the manufacturer's protocol (SYBR Select Master Mix, Applied Biosystems) on 7500 Fast Real-Time PCR System instrument (Applied Biosystems). A single PCR reaction included a 1 μL portion of the reverse transcription product. Oligonucleotide primers were selected to detect markers of the immune system response [activating transcription factor 3 (*ATF3)*, interleukin 8 (*IL-8*), interleukin 10 (*IL-10*), interleukin 1β (*IL-1β*), interleukin 6 (*IL-6*), macrophage receptor MARCO (*MARCO*) and tumor necrosis factor (*TNFα*)]. Additionally, gene expression profiles of IL-8 receptors, C-X-C motif chemokine receptor 1 (*CXCR1*) and C-X-C motif chemokine receptor 2 (*CXCR2*), were investigated. The details are listed in Table 1. To demonstrate the presence of PACAP receptors in zebrafish neutrophils, we have created specific oligonucleotide primers using Primer-BLAST tool. PAC1 is encoded by *adcyap1r1a* and *adcyap1r1b* genes. VPAC1 is encoded by *vipr1a* and *vipr1b* genes. VPAC2 is encoded by *vipr2* gene. The details are listed in Table 1. In both cases, *β-actin* was used as a house-keeping gene. Each sample of PCR product was analyzed against a *β-actin* control to standardize the results. The following PCR protocol was used with a 7500 Fast Real-Time PCR System instrument (Applied Biosystems): denaturation for 10 min at 95°C followed by 40 cycles of 15 s at 95°C, 1 min at 60°C and 15 s at 95°C. The results regarding markers of the immune system response are presented as relative quantities (RQ) of mRNA which were analyzed using the comparative Ct method. The values of gene expression of PACAP receptors were calculated in each group as a relative expression to *β-actin*. Each sample was analyzed in triplicate in three separate experiments.

**Table 1:** Primers used in the study.

Gene	Forward 5’-3’	Reverse 5’-3’	Source/Accession no.
*ATF3*	CCGTCAGAGATCAGTGCGTCAGCTTTG	GTTCTGAGCGCGGACGATGCAGGTGG	[27]
*IL-1β*	GAACAGAATGAAGCACATCAAACC	ACGGCACTGAATCCACCAC	[28]
*IL-6*	TCAACTTCTCCAGCGTGATG	TCTTTCCCTCTTTTCCTCCTG	[28]
*IL-8*	TGTGTTATTGTTTTCCTGGCATTTC	GCGACAGCGTGGATCTACAG	[28]
*IL-10*	GGAGACCATTCTGCCAACAGC	TCTTGCATTTCACCATATCCCG	[28]
*MARCO*	AGCCAAGGGGTAAAAGGAGAC	TTGGTCCAGGTGAGCCTTTTC	[29]
*TNFα*	ACCAGGCCTTTTCTTCAGGT	GCATGGCTCATAAGCACTTGTT	[30]
*CXCR1*	TTCAGTTCGGCTGCACTATG	GGAGCAACTGCAGAAACCTC	[31]
*CXCR2*	TGACCTGCTTTTTTCCCTCACT	TGACCGGCGTGGAGGTA	[31]
*adcyap1r1a*	GAGTTGGACGGTGAACAGGT	GGCTGGACATGCGTATCTGA	NM_001142926.1 NM_001142925.1 NM_001013444.2
*adcyap1r1b*	GATGATCCCAACAGTGAACCG	ACAGGGCATCCAGACAACTTGA	XM_677888.7
*vipr1a*	CTGAAGGCGGTGGCAGTAAT	TTGCAGCCCACAGATCCATAG	XM_021467142.1 XM_002660797.4
*vipr1b*	TGCACTCGCAACTACATCCA	AGCCAACAGAACCAGTGGAG	NM_001013353.1
*vipr2*	GGATCCTTTCAGGGCTTGGT	GAGGAACTGTGCAGACGGTA	NM_131779.1
*β-actin*	CGAGCAGGAGATGGGAACC	CAACGGAAACGCTCATTGC	[32]

### Statistical analysis

The statistical analysis was performed using GraphPad Prism, version 5.0. Data with Gaussian assumption were analyzed using a one-way ANOVA test and a one-way analysis of variance with Tukey multiple comparisons tests as a post test. Data analyses not assuming Gaussian distribution were based on a one-way ANOVA test and a Kruskal–Wallis test with Dunn’s multiple comparisons test as a post test. The error bars represent the mean ± SEM. The significance level was set at α = 0.05 (95% confidence intervals).

## Results

### Inhibitory effects of PACAP-38 on neutrophil migration towards damaged hair cells

**Fig 2:**
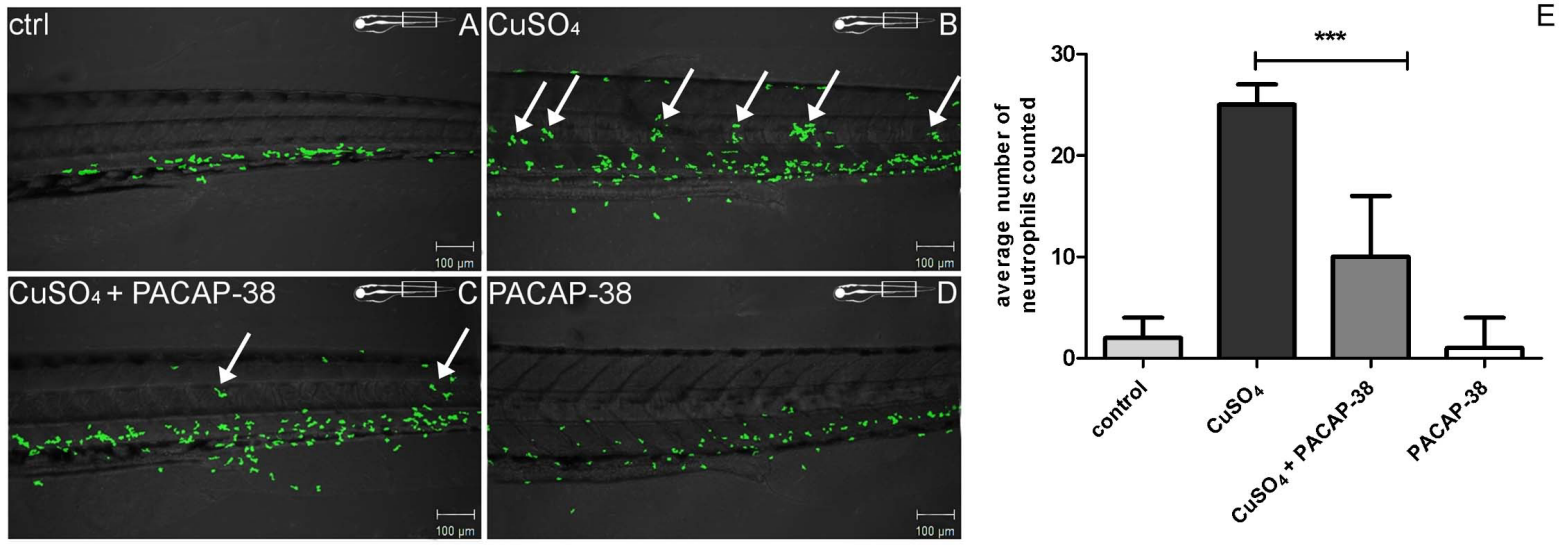
A set of microphotographs and a graph documenting inhibition of neutrophil migration towards 10 µM CuSO_4_ exposed hair cells resulting from co-treatment of 5 dpf Tg(MPX:GFP) zebrafish larvae (exhibiting green fluorescence in neutrophils) with 100 nM PACAP-38. (A) The control untreated larva presented normal distribution of neutrophils which were found in the ventral myotomes of the trunk and tail. (B) 10 µM CuSO_4_ exposure evoked the migration of the immune cells towards the midline of the body and the formation of characteristic concentrations very close to, and around, the neuromasts (arrows). (C) 100 nM PACAP-38 co-treatment resulted in the inhibition of the neutrophil migration, which was reflected by a decreased number, or complete lack of, the green fluorescent cells in the area of natural neuromast localization (arrows). (D) 100 nM PACAP-38 itself did not visibly alter the natural distribution of neutrophils. The visualization was accomplished using a Zeiss LSM-700 confocal microscope. (E) The graph presenting the influence of 100 nM PACAP-38 on the number of the neutrophils concentrated around right posterior lateral line neuromasts (PLL) (L1, LII.1, L2, LII.2, L3, L4, L5 and L6) after 10 µM CuSO_4_ exposure. The presented values refer to the average number of neutrophils in each group. 100 nM PACAP-38 treatment resulted in a significant, over two-fold decrease in the number of the neutrophils found singly in the area defined by notochord borders and those associated with neuromasts as compared to that determined in the 10 µM CuSO_4_-exposed group (one-way ANOVA, Kruskal–Wallis test with Dunn’s post-test, GraphPad Prism 5, *p* < 0.001). N/group = 15.

In the control larvae, most of the immune cells remained in the area of ventral myotomes (Fig 2A). In the animals treated with PACAP-38 only, the distribution of the cells was very similar to that in the control group, suggesting that PACAP-38 itself had no visible influence on the behavior of neutrophils (Fig 2D). In contrast, in the individuals exposed to 10 µM CuSO_4_ for 40 min, the immune cells were dispersed throughout the body, forming characteristic clusters in the midline (Fig 2B). The distribution of the clusters matched that of neuromasts, suggesting that the migration of neutrophils was associated with, and directed towards, damaged hair cells (Fig 2B). In contrast to the copper exposure, co-treatment with PACAP-38 inhibited the migration of neutrophils and resulted in an over 2-fold decrease in the number of the immune cells, both those associated with the neuromasts and single ones found in the area defined by notochord borders (Fig 2C and 2E). Data are also provided as supporting information (S2 File).

### Effects of PACAP-38 on mRNA expression level of pro-inflammatory and stress-inducible genes

**Fig 3:**
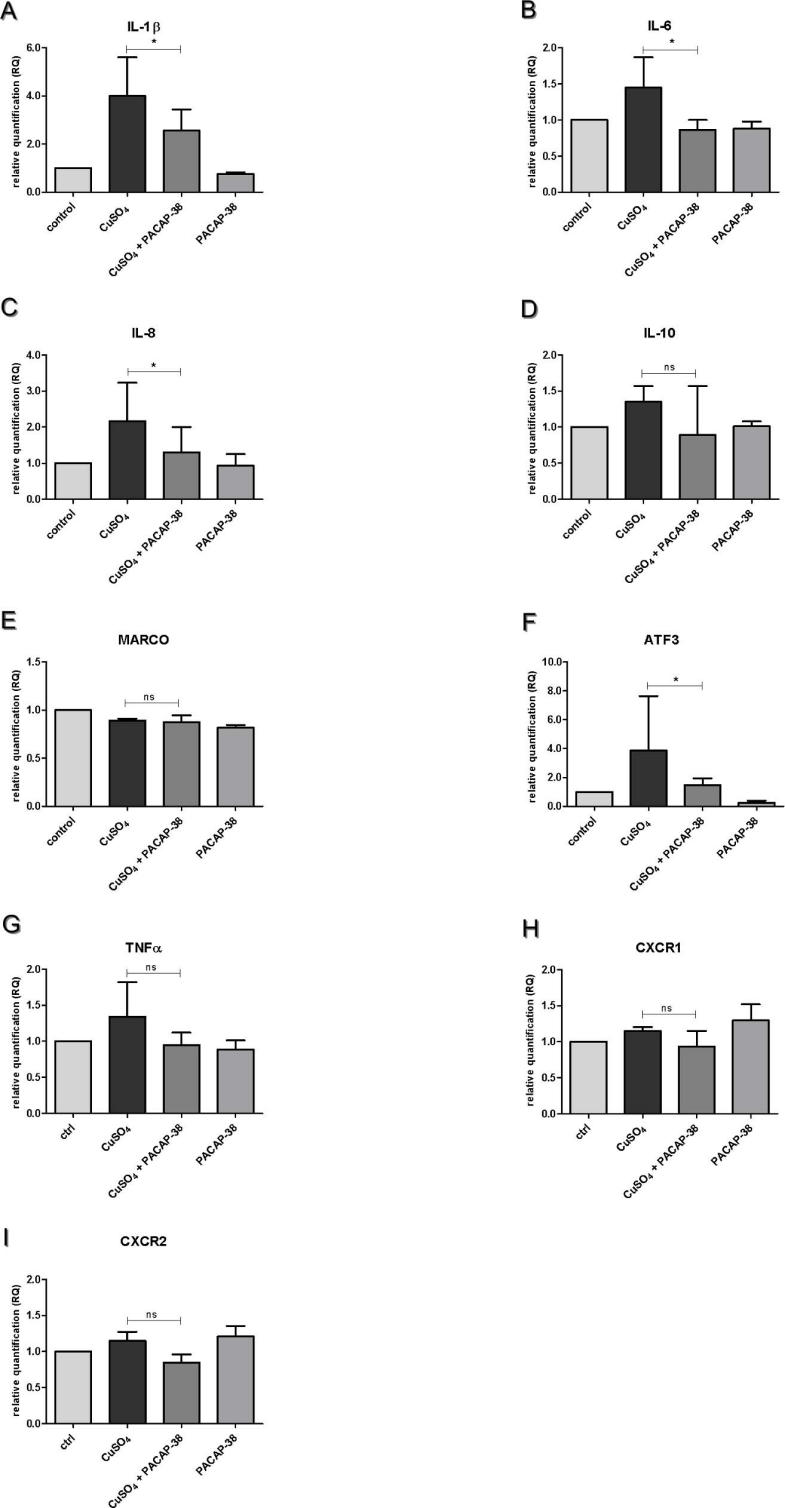
Expression profiles of pro-inflammatory and stress-inducible genes. The graphs present the mRNA expression of (A) interleukin 1β (*IL-1β*), (B) interleukin 6 (*IL-6*), (C) interleukin 8 (*IL-8*), (D) interleukin 10 (*IL-10*), (E) macrophage receptor MARCO (*MARCO*), (F) activating transcription factor 3 (*ATF3*), (G) tumor necrosis factor (*TNFα*), (H) C-X-C motif chemokine receptor 1 (*CXCR1*) and (I) C-X-C motif chemokine receptor 2 (*CXCR2*) from pooled (n = 30) 5dpf wild-type zebrafish larvae in four experimental groups: 1) control, 2) exposed to 10µM CuSO_4_ for 40 min, 3) exposed to a mixture of 100 nM PACAP-38 + 10µM CuSO_4_ for 40 min preceded by one hour pre-incubation with 100 nM PACAP-38 only, and 4) exposed to 100nM PACAP-38 only. Each group was covered by samples analyzed in triplicate in three separate experiments. Data in the figure represent the average of the three individual experiments. Gene expression values were normalized to housekeeping gene *β-actin*. To maintain image clarity, only differences between CuSO_4_ exposed and PACAP-38 co-treated groups are marked on the graphs. Co-treatment with PACAP-38 significantly reduced up-regulated by copper treatment *IL-1β* and *IL-6*, *IL-8* and *ATF3* gene expressions (A, B, C and F) (one-way ANOVA, Kruskal–Wallis test with Dunn’s posttest, GraphPad Prism 5, *p* < 0.05), while it had no significant influence on *IL-10*, *MARCO* or *TNFα* genes (D, E and G) (one-way ANOVA, Kruskal–Wallis test with Dunn’s posttest, GraphPad Prism 5, *p* > 0.05). With regard to *CXCR1* and *CXCR2*, 10 µM copper chemical injury and 100 nM PACAP-38 treatment did not significantly change the expression level of the receptors. (one-way ANOVA, Kruskal–Wallis test with Dunn’s post-test, GraphPad Prism 5, *p* > 0.05).

The effects of 100 nM PACAP-38, 10 µM CuSO_4_ and mixture of 100 nM PACAP-38 and 10 µM CuSO_4_ exposure on the mRNA levels of various genes (*ATF3*, *IL-1β*, *IL-6*, *IL-8*, *IL-10*, *TNFα*, *MARCO*, *CXCR1* and *CXCR2*) encoding pro- and anti-inflammatory factors were determined by RT-qPCR (Fig 3A–3I, S3 File). The expression profiles of all genes examined remained statistically unchanged after PACAP-38 treatment only (p > 0.05) (Fig 3A–3I). Copper treatment resulted in up-regulation of *IL-1β*, *IL-6 IL-8*, and *ATF3* (p < 0.01) (Fig 3A, 3B, 3C and 3F), while the expression of *IL-10*, *MARCO* and *TNFα* remained statistically unchanged (Fig 3D, 3E and 3G). In groups exposed to 10 µM CuSO_4,_ co-treatment with 100 nM PACAP-38 resulted in a statistically significant decrease in the expression of previously up-regulated *IL-1β*, *IL-6 IL-8*, and *ATF3* (p < 0.05) (Fig 3A, 3B, 3C and 3F). Although expression levels of *IL-10* and *TNFα* did not demonstrate any statistically significant differences between the experimental groups, some tendency was clearly visible. They were raised after administration of copper but decreased after co-treatment with 100 nM PACAP-38 (Fig 3D and 3G). To ascertain the involvement of receptors for IL-8 in neutrophil migration towards damaged lateral line hair cells during copper and PACAP-38 co-treatment, the expression levels of *CXCR1* and *CXCR2* were determined. Although 40 min exposure to 10 µM copper solution was enough to increase the expression level of *IL-8*, it did not change the transcriptional levels of both *CXCR1* and *CXCR2*. The addition of 100 nM PACAP-38 also did not alter them. (Fig 3H and 3I) (one-way ANOVA, Kruskal–Wallis test with Dunn’s posttest, GraphPad Prism 5, p > 0.05).

### Effects of PACAP-38 on hair cell necrosis

**Fig 4:**
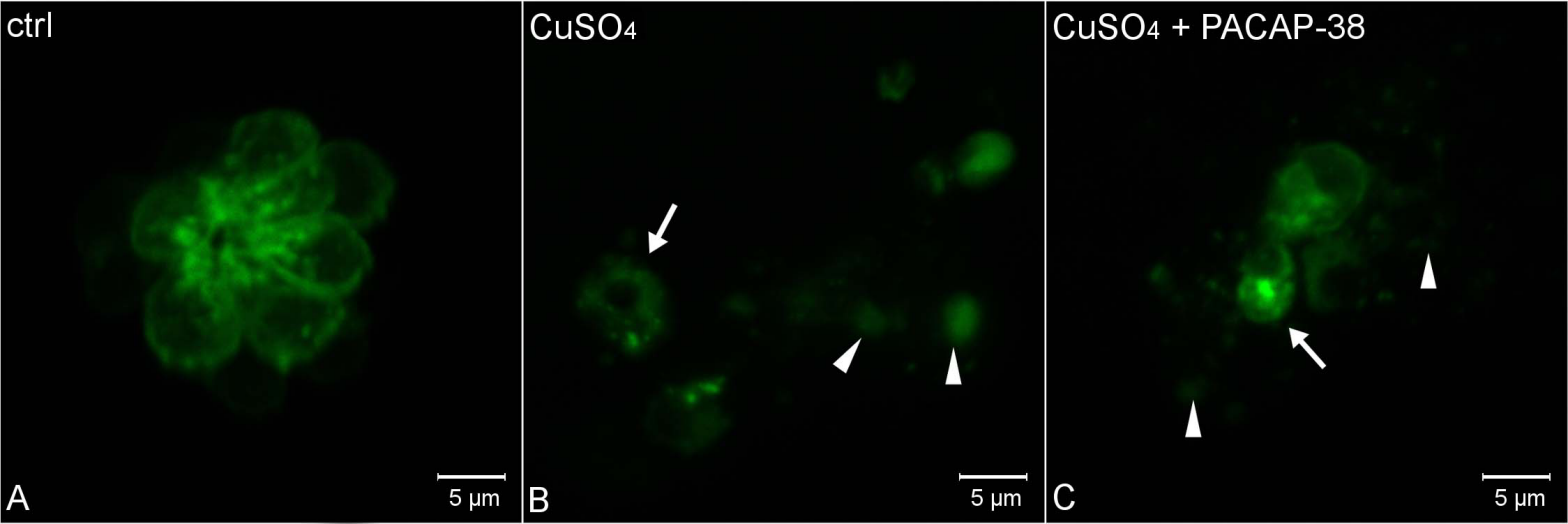
Morphology of L2 neuromast hair cells in 5 dpf Tg(pou4f3:GAP-GFP) zebrafish larvae. (A) control, (B) exposed to 10 µM CuSO4 for 40 min, and (C) exposed to the mixture of 10 µM CuSO_4_ and 100 nM PACAP-38 for 40 min, following 1 hour pre-incubation with 100 nM PACAP-38 only (n/group = 15). The visualization was accomplished using a Zeiss LSM-700 confocal microscope. (A) Hair cells in the control group exhibited morphology without any necrosis features. (B) Copper exposure evoked severe necrosis, resulting in hair cell rosette disintegration. Hair cells were round-shaped and swollen (arrowheads). Other groups of hair cells appeared shrunken and fragmented (arrow), suggesting the involvement of other death pathways. (C) In the PACAP-38 co-treated group, the necrosis was comparably severe. The same necrotic signs, i.e. round-shaped and swollen (arrow heads), as well as shrunken and fragmented (arrow) cells were also observed.

The control hair cells showed normal and regular pear-shaped morphology (Fig 4A). 10 µM CuSO_4_ evoked rapid and acute necrosis of hair cells in PLL (Fig 4B). The hair cell rosette was totally destroyed and nearly all of the cells demonstrated an abnormal shape. They became round-shaped, swollen (Fig 4B, arrow heads) and presented far-reaching dilapidation. Moreover, the cell membrane was ruptured and released the cell content, thus the pictures taken make an impression of being out of focus. Some hair cells appeared shrunken and fragmented, presumably presenting other death pathways (Fig 4B, arrow). In turn, PACAP-38 did not prevent hair cells from necrosis. In the 100 nM PACAP-38 co-treated group, hair cells remained necrotic and demonstrated all the regressive features (Fig 4C).

### Expression profile of genes encoding PACAP receptors on neutrophils

**Fig 5:**
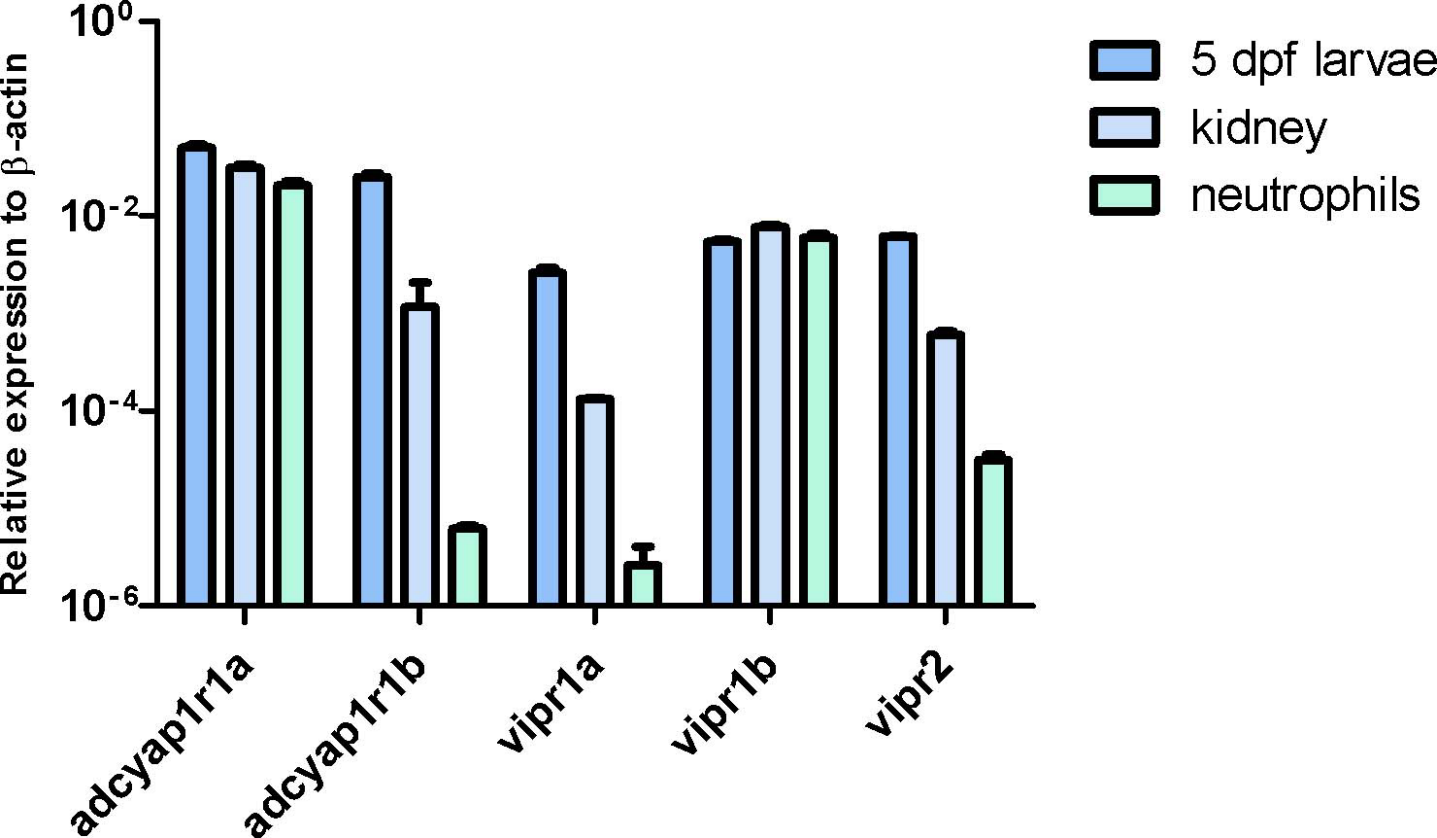
A graph illustrating expression profiles of genes encoding three PACAP receptors (PAC1, VPAC1 and VPAC2) in samples from zebrafish 5 dpf larvae, kidney tissue and neutrophils. The material concerning larvae consisted of pooled individuals (n = 30), the kidney tissue refers to a single cell suspension derived from kidneys of five adult zebrafish and the neutrophil population consisting of 500,000 GFP+ sorted cells by FACS. Each collection was covered by samples analyzed in triplicate. Data in the figure represent the average of the representative experiment. Gene expression values were normalized to housekeeping gene β-actin. The existence of all types of PACAP receptor transcripts was reported in each collection, however at a different levels. In neutrophils, *adcyap1r1a* appeared as a predominant form for PAC1 and, for the VPAC family, the most prevalent was *vipr1b*. The lowest expression level was exhibited by *adcyap1r1b* and *vipr1a* genes. Interestingly, *adcyap1r1a* and *vipr1b* were expressed at similar levels in each studied sample, whereas for the remaining PACAP receptor genes, the highest expression levels were found in whole larvae, lower in the kidney tissue and the lowest was in neutrophils.

The expression level of genes for PACAP receptors was investigated quantitatively by Real-Time PCR. In vertebrates, the molecular cloning of PACAP receptors has shown the existence of three distinct receptors: one that recognizes PACAP specifically—PAC1, and two binding PACAP and VIP equally firmly—VPAC1 and VPAC2 [33]. Since compared to other vertebrates, the zebrafish genome is duplicated [34], we aimed to investigate the following genes encoding PACAP receptors: *adcyap1r1a*, *adcyap1r1b*, *vipr1a*, *vipr1b* and *vipr2*. Basically, we found the presence of mRNA for all types of PACAP receptors in the neutrophils. However, *adcyap1r1a* appeared to be a predominant form for PAC1, while for VPAC family, the most prevalent was *vipr1b*. The lowest expression levels were exhibited by *adcyap1r1b* and *vipr1a* genes. In the larvae and kidney tissue, all genes encoding PACAP receptors showed significant, but differentiated, expression. It was found that, as in neutrophils, the highest expression level in the kidney tissue and larvae was exhibited by *adcyap1r1a*. However, unlike neutrophils, in the kidney tissue and whole larvae, *adcyap1r1b* was also expressed at a relatively high level. Concerning VIP receptors, *vipr1a* showed the lowest expression level not only in neutrophils, but also in the kidney tissue as well as the whole larvae. Interestingly, *adcyap1r1a* and *vipr1b* were expressed at similar levels in each sample investigated, whereas for the remaining PACAP receptor genes, the highest expression levels were found in whole larvae, lower in the kidney tissue and the lowest was in neutrophils. The respective data are presented in Fig 5 and S4 File.

## Discussion

The present study provides, for the first time, clear evidence that PACAP-38 inhibits the migration of neutrophils towards inflamed hair cells in the zebrafish. In terms of the usage of zebrafish in studies concerning the auditory system, it is extremely important that their inner ear resembles that found in higher vertebrates developmentally and anatomically, although does not possess structures corresponding with the mammalian outer and middle ear [35]. Besides the ability to regenerate [36–39], hair cells in the zebrafish inner ear and lateral line are structurally, functionally and molecularly similar to mammalian ones [22]. It should be emphasized that these cells are considered to be one of the most crucial elements for normal hearing and vestibular function. The idea of using zebrafish for investigating typical anti-inflammatory drugs or immune response in relation to hair cells has already been introduced [24]. However, no information is available on the involvement of neuropeptides in these immune relationships. Previously, we have demonstrated that 100 nM PACAP-38 prevents zebrafish hair cells from oxidative stress-induced apoptosis [6]. Because many factors and signaling pathways that are activated by inflammation are also involved in the regulation of cell apoptosis [23], we considered PACAP-38 to be a promising candidate for ameliorating inflammation associated with oxidative stress-damaged zebrafish hair cells. PACAP-38 is well known for its involvement in inflammation and immunity [7,40,41]. The majority of studies have reported that PACAP-38 reduces inflammatory changes, however, there are some data revealing its reverse properties, such as vasodilation and edema formation in the rabbit eye [42]. Although the anti-inflammatory effect of PACAP-38 has been shown in several tissues and organs, such as airways [43], the central nervous system [44,45] or joints [46], there is no evidence for its action in the auditory system.

In the present study, it was demonstrated that the administration of 100 nM PACAP-38 resulted in an over 2-fold decrease in the formerly increased number of neutrophils in the PLL. It was earlier found that the dose of CuSO_4_ applied in this study evoked severe necrosis to zebrafish hair cells after only a 5 minute exposure [47]. Necrotic cell death is typically connected with some pathological events and stimulates a rapid inflammatory response. The question is whether the reported inhibition of neutrophil migration in PACAP-38 co-treated animals results from anti-necrotic ability of the peptide or its direct inhibitory influence on the inflammation process. The anti-apoptotic properties of PACAP-38 are well known and are broadly described in the literature [1,2,4,6,48,49], but its action against necrosis is poorly discussed. The current studies revealed that PACAP-38 does not prevent hair cells from rapid or extensive necrosis, despite the fact that there is some evidence for its ability to inhibit a specific type of necrosis—oncosis in mice [50]. Thus, we can conclude that in the current study, PACAP-38 affected only the immune system cells. The next issue is exactly how PACAP-38 regulates the immune response to the damaged hair cells. It is possible that the peptide inhibits the migration of neutrophils, altering their behavior via direct or indirect mechanisms. It has been already demonstrated that PACAP-38 differently modulates the behavior of immune cells. The current results corroborate findings obtained by Kinhult et al. (2001) [13], who found that PACAP-38 inhibited fMLP-induced human neutrophil chemotaxis. Similarly, PACAP-38 has an inhibitory effect on lymphocyte chemotaxis [51,52], but stimulates the chemotaxis of macrophages [52]. This is more evidence that PACAP-38 can both promote and inhibit immune responses. It is also possible that PACAP-38 acts directly in the place where inflammation occurs. Based on the fact that chemical inhibition of NADPH oxidase significantly decreased the leukocyte migration towards neuromasts, it was concluded that for leukocyte recruitment, formation of a reactive oxygen species (ROS) gradient upon copper-induced inflammation is a critical step [24]. The relationship between PACAP-38 and NADPH oxidase has already been recognized, because PACAP-38 neuroprotective effect was achieved by inhibition of NADPH oxidase and consequent reduction of microglia-derived ROS [53]. Moreover, as PACAP-38 plays an anti-oxidant role [54], it could be possible that it decreases ROS formation in the hair cells, thus leading to restraining the inflammatory process.

If PACAP-38 inhibit the migration of neutrophils via a direct mechanism, this could be accomplished through receptors on their surface. In the present study, the existence of all transcripts for PACAP receptors in zebrafish neutrophils obtained from the renal tissue were comprehensively demonstrated for the first time. It should be mentioned, that in fish, the immune organ constituting the main source for neutrophils is the kidney [55]. Our findings demonstrate which receptors and which genes are expressed specifically in zebrafish neutrophils. Considering the PACAP-specific receptor PAC1, we have found relatively high expression of *adcyap1r1a*, at almost the same level as that in the kidney tissue and whole larvae, which suggests its involvement in the studied process. Next to the *adcyap1r1a*, the most expressed was *vipr1b*. Its expression level was also comparative with that in the kidney tissue and the whole larvae. This interesting finding provides new insight into a role of VIP in the regulation of neutrophil behavior in the zebrafish. However, a study by Kinhult et al. (2002) [16] performed in humans has revealed that only PACAP, not VIP, functions as an activator of neutrophils. The remaining genes encoding PACAP receptors in neutrophils were expressed at relatively low levels compared to those found in the kidney tissue. Since the isolated fraction of neutrophils was slightly (by 2%) contaminated with the renal tissue, the expression values determined in this fraction might be due to other renal cellular elements, such as tubule cells [56] or vasculature [57]. Nevertheless, the current *in vivo* results corroborate the *in vitro* findings of Kinhult at al. (2002) [16] and demonstrate that PACAP-38 can directly affect neutrophils.

The current *in vivo* investigations were supplemented with Real-Time PCR analysis, which clearly confirmed that the effects observed could have a molecular basis at the level of gene expression. The copper treatment increased the expression of *IL-1β*, *IL-6*, *ATF3* and *IL-8*. The mRNA expression levels of four typical stress-inducible markers, *IL-1β*, *IL-6*, *IL-8* and *ATF3*, were significantly decreased after PACAP-38 treatment. This is the first evidence of PACAP-38 regulation towards *ATF3*. ATF3 was considered because it was described recently as a novel regulator of neutrophil migration in mice [58]. Earlier comprehensive studies [59] found that *ATF3* is an inducible adaptive response gene encoding the ATF/CREB family of transcription factors. After copper exposure, its expression level increased over 4-fold. Although ATF3 is generally thought to promote apoptosis and cell cycle arrest [59], its role in immune regulation is also recognized [60]. On one hand, ATF3 is thought to be induced by signals such as pro-inflammatory cytokines [60], whereas with the use of ATF3 null mice it was demonstrated that ATF3 inhibits *IL-6* and *IL-12b* transcription by altering chromatin structure [61]. Moreover, in the present study, copper exposure also caused a significant upregulation of two pro-inflammatory cytokines, *IL-1β* and *IL-6*. At this stage, it is hard to conclude if there is any correlation between *ATF3* and the investigated cytokines. An inhibitory role towards *IL-6* gene expression is already well recognized [62], therefore, the results of the current study confirm this property in other experimental conditions. Since the influence of a nanomolar dose of PACAP-38 on the expression of *IL-1β* was observed for the first time, the functional basis of this process needs to be further investigated. On the other hand, there is some interesting information on the mutual relationship between PACAP-38 and IL-1β in rats. These substances have been found to synergistically stimulate IL-6 secretion from astrocytes [63] and IL-1β intraperitoneal injections stimulate PACAP mRNA expression in neurons [64]. IL-1β is a proinflammatory cytokine facilitating the activation of neutrophils. Therefore, it can be speculated that its down-regulation could be involved in the inhibition of neutrophil migration to damaged hair cells observed in the present study. However, because IL-1β strongly upregulates IL-6 protein secretion [65], the effect of PACAP-38 on *IL-6* found in the current study could be indirect and may be mediated by *IL-1β*. Moreover, with the use of Real-Time analysis, strong evidence was gained that PACAP-38 decreased the expression of *IL-8* gene formerly upregulated by copper exposure. It should be noted that some previous data (agreeing with the current data) also reveal PACAP-38 inhibitory properties towards *IL-8* [62]. IL-8 is known for its involvement in neutrophil attraction and activation. Based on the current investigations, it could be stated that one of the possibilities of PACAP-38 action towards inhibiting neutrophil migration is modulation of the production of chemotactic factors, in this case IL-8. However, the gene expression levels of IL-8 receptors *CXCR1* and *CXCR2* remained unchanged after both copper exposure and PACAP-38 treatment. Although the expression level of *IL-10* did not demonstrate statistically significant differences between the groups, its fluctuations revealed a certain characteristic tendency. In general, it could be said that it was raised after the administration of copper and was decreased after co-treatment with PACAP-38. Conversely, previous data have revealed a PACAP-38 stimulatory effect towards *IL-10* [66]. The gene expression of another investigated cytokine, TNFα, was significantly increased after copper treatment, but it was insignificantly inhibited by PACAP-38. Nevertheless, it can be concluded that the current observations fully correspond with common data presenting PACAP-38 as an inhibitory factor in TNFα production [67–69]. MARCO is a macrophage receptor belonging to the class A of the scavenger receptor family and is an element of the innate antimicrobial immune system. In the present study, the expression level of *MARCO* remained unchanged after PACAP-38 treatment and its role as an antimicrobial agent has been investigated and revealed only by Hannibal et al. (1999) [64]. These authors found that administration to rat of bacterial lipopolysaccharide (LPS) resulted in a marked increase in both PACAP-38 immunoreactivity and its gene expression. Furthermore, Bik et al. (2006) [70] revealed that in LPS-induced acute inflammation PACAP-38 exerted a short-term modulation of immune and endocrine response.

The inner ear, including structures covered with the sensory epithelium, was previously assumed to be a putative “immune-privileged” organ, like the brain or eye, due to the existence of its tight junction-based blood-labyrinth barrier [71] which blocks immune cells, inner ear antigens and antibodies. However, recent studies have demonstrated that the cochlea is a common site of inflammation and, under pathological conditions, contains a characteristic “mononuclear phagocyte system” in the spiral ligament [72] and “perivascular macrophage-like melanocytes (PVM/Ms)” distributed in the stria vascularis [73]. Additionally, the murine inner ear systemically immunized with LPS antigen revealed enhanced leukocyte infiltration and cochlear IL-1β expression [74]. Furthermore, *in vivo* production of TNF-α, IL-1β, and IL-6 in the murine cochlea was reported after immunization with keyhole limpet hemocyanin (KLH) [75]. These observations seem to be a clear confirmation that the inner ear is prone to immune-mediated disorders and, together with the current results, suggest the presence of a local immune system in the inner ear structures.

Unequivocal interpretation of the physiological significance of the present results is difficult or even impossible. Generally, this is due to potential ambiguities regarding the biological implications of inflammatory responses. It is well known that inflammation is an integral part of the majority of pathological processes. Depending on numerous external and internal factors, such as the type of affected tissue, nature of pathological stimuli or even the type of species of the host, inflammatory response can have beneficial (for instance, reparative), or, conversely, harmful consequences (if it lasts too long). Therefore, anti-inflammatory drugs should be applied with particular care. Considering the above explanations, it seems to be inappropriate to consider the present results in “beneficial/detrimental” perspective. We can only say that PACAP-38 is able to inhibit migration of neutrophils and decreases the gene expression of some cytokines, which is an important phenomenon occurring in the course of inflammatory response. Accordingly, it should be taken into consideration as a substance through which modulatory actions towards the intensity of inflammation affecting structures of the inner ear (also mammalian) could be accomplished.

In conclusion, the present study has revealed that a nanomolar dose of PACAP-38 inhibits neutrophil migration towards damaged zebrafish hair cells undergoing chemically induced necrosis and consequent inflammation. As neutrophils are critically involved in the inflammation process, their decreased number (or complete lack) around the damaged neuromasts in the presence of PACAP-38 seem to be a clear evidence that this substance has an anti-inflammatory role in at least tissues investigated. Moreover, PACAP-38 co-treatment resulted in a considerable decrease in the expression level of *ATF3*, *IL-8*, *IL-1β* and *IL-6* upregulated after copper exposure. This hopefully provides new insights into the molecular mechanisms of the PACAP-38 anti-inflammatory effect. It seems that studies considering the structures of the auditory system carried out on zebrafish may be fully related to those performed in mammals. The present findings seem to support this statement and suggest that PACAP-38 plays an important regulatory role in the inflammatory response associated with pathological processes affecting hair cells and it cannot be excluded that this property of the peptide has a more universal significance, not only limited to lower vertebrate species.

## Supporting information

S1 File Illustrations of the settings used on FACS. (A) Forward (FSC) and side scatter (SSC) plot. This measurement is related to designating and identifying cells according to their size and internal granularity or complexity of a particle, respectively. The R5 gate was set so as to exclude cellular debris and imperfectly isolated cells. (B) A histogram was prepared illustrating kidney tissue cells separated according to the GFP fluorescence intensity (GFP FITC-A). The histogram demonstrates two peaks demarcating GFP- and GFP+ cells. GFP-positive cells constituted 16% of the cell suspension.
(TIF)

S2 File Raw data of neutrophil counting. (XLSX)

S3 File Raw data of Real-Time PCR results respecting pro-inflammatory and stress-inducible genes. (XLSX)

S4 File Raw data of Real-Time PCR results respecting PACAP receptors. (XLSX)
