# Supplementary figures and images for: Pituitary adenylate cyclase–activating polypeptide (PACAP-38) plays an inhibitory role against inflammation induced by chemical damage to zebrafish hair cells
Source: PLoS One. 2018 Jun 1;13(6):e0198180. doi: 10.1371/journal.pone.0198180 (PMC5983416; doi:10.1371/journal.pone.0198180)

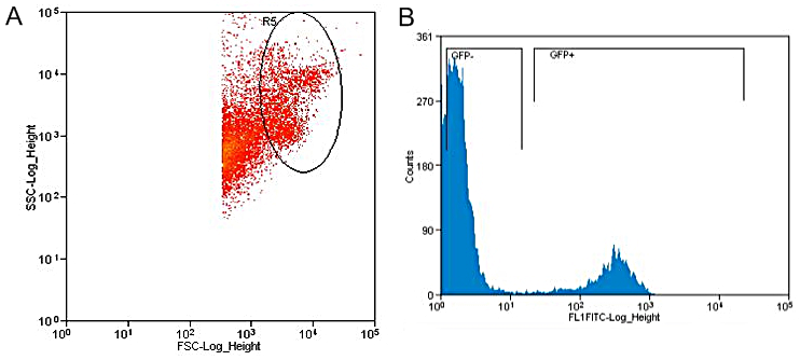

Supplement: S1 File — (A) Forward (FSC) and side scatter (SSC) plot. This measurement is related to designating and identifying cells according to their size and internal granularity or complexity of a particle, respectively. The R5 gate was set so as to exclude cellular debris and imperfectly isolated cells. (B) A histogram was prepared illustrating kidney tissue cells separated according to the GFP fluorescence intensity (GFP FITC-A). The histogram demonstrates two peaks demarcating GFP- and GFP+ cells. GFP-positive cells constituted 16% of the cell suspension. (TIF) [file pone.0198180.s001.tif]
